# Clinical Lecture on Laryngeal and Throat Affections, Delivered at King’s College Hospital

**Published:** 1853-04

**Authors:** Robert B. Todd

**Affiliations:** Physician to the Hospital


### RECORD OF MEDICAL SCIENCE.

### PATHOLOGY AND PRACTICE OF MEDICINE.

*Clinical Lecture on Laryngeal and Throat Affections, delivered at King's College Hospital.* By Robert B. Todd, M. D. F. R. S., Physician to the Hospital.

Gentlemen,—You have lately had the opportunity of observing two cases, of the disease of the larynx, the other of an affection of the mucous membrane of the fauces, which will enable me to bring these subjects under your notice to-day.

Let me first remark, with respect to laryngeal diseases in general, whether it be acute or chronic, that it is very much influenced by diathesis, both in its origin and in its duration. This is very manifest in the case of the strumous as well as of the gouty diathesis. Persons of either of these forms of constitution, when once they have been attacked with laryngeal inflammation, find it very difficult, sometimes, indeed, impossible, to shake off the disease.

One of the most formidable of the acute affections of the larynx, happily less frequently met with now than formerly, is the inflammatory or membranous croup,—a disease which is characterised by the rapid formation of a layer of coagulable lymph, forming a false membrane, that moulds itself to the interior of the larynx, and will extend down the trachea, whence it is sometimes called "cynanche trachealis," even into the bronchial tubes. The pathology of this disease is not as yet by any means settled; but it may, I think, be said, that the true membranous croup is, of all laryngeal diseases, the least associated with peculiarity of diathesis. Why it is in so marked a manner a disease of childhood has received no explanation. We have in adult life a disease somewhat analogous to it, although affecting the pharyngeal rather than the laryngeal membrane. I mean that disease which is accompanied by a membranous exudation on the mucuous membrane of the velum and back of the pharynx, which the French have designated *diphtherite* —a malady in close alliance with erysipelas. Can it be that the cause and the pathology of diphtherite and of croup are alike? This subject is one which demands careful investigation, and the more so as the results of our ordinary means of treating croup are far from being satisfactory.

One of the most common forms of laryngeal disease is that which is connected with the strumous or tubercular diathesis—this is also known as laryngeal phthisis—it is usually associated with tubercular deposits in the lungs. That form of cachexia which is induced by the syphilitic poison will also often give rise to laryngeal disease, generally chronic, but sometimes exhibiting very acute and urgent symptoms. These two forms of chronic laryngeal disease may be confounded the one with the other.

Erysipelas may affect the larynx, and give rise to the most serious consequences. It is well known that the erysipelatous poison is very prone to attack the mucous membrane of the fauces. From that the erysipelas may spread either forwards through the nostrils to the face and head, or backwards and downwards to the larynx. Erysipelas of the larynx is apt to induce a rapidly œdematous condition of the submucous areolar tissue, giving rise to that fearful malady, acute œdema of the glottis, by which the chink, so important to life, is very quickly encroached upon, and the difficulties of a severe and rapid dyspnœa, superadded to the depressing influence of the erysipelatous poison, speedily destroy life.

To these affections I may add an inflammatory condition of a chronic form, not destructive to life, nor to the tissues of the larynx. It is a chronic inflammation of the mucous membrane, very often described as a relaxed condition, with considerable enlargement of the mucous follicles. This affection is often connected with the lythic or gouty diathesis, and it likewise frequently occurs in debilitated states of the system from various causes. It is sometimes associated with a peculiar state of the nervous system, a form of hypochondriasis. That condition of throat which is so apt to occur in clergymen is of this kind.

I shall illustrate to-day the tubercular affection of the larynx, and that relaxed condition of its mucous membrane to which I have last referred.

The first case which I shall bring under your notice affords a good example of disease of the larynx occurring in the tubercular diathesis.

The subject of this affection was a girl of the name of Reynolds, in Lonsdale Ward, (Vol. xxxiv., p. 160;) she was 18 years of age, and of delicate health. The history of her case afforded abundant evidence of the existence of phthsis in her family, as she had lost her mother and one sister by this disease.

The affection from which she was suffering has been badly named "laryngeal phthsis," because the name would lead you to suppose that the disease was limited to the larynx; whereas, I believe, it never occurs without the presence of tubercles in the lungs, either in the crude or softened state. In some cases, the laryngeal symptoms are the first to manifest themselves. A patient having indications of a phthisical tendency, is found on inquiry, before any symptoms of tubercle had manifested themselves, to have been the subject of frequent slight affections of the larynx, accompanied by hoarseness and cough, and attributed to exposure to changes of temperature.

In other cases, the symptoms of phthisis develope themselves before the laryngeal symptoms commence. In the present instance, however, the affection of the larynx appeared first, and upon superficial examination at an early period, the diseases might have been viewed as one of laryngitis simply.

The patient told us that, in November last, soon after exposure to the wet and cold, she became troubled with a feeling of soreness about the throat, which was followed by hoarseness and loss of voice, and, at the same time, she became affected with a dry, suffocating cough, accompanied by severe pain in the region of the larnyx.

Pain referred to the larynx is one of the most constant symptoms of the disease, and will rarely be found entirely absent. Usually the pain causes great distress to the patient. The affection of voice varies according to the seat of the disease. If the epiglottis and adjacent folds of membrane only are involved, the voice will probably not suffer much; but, if the inflammation extend downwards, the affection of the voice will vary in severity according to the extent to which the ventricles of the larynx or the vocal cords are involved.

A symptom soon appeared in our patient which must always be regarded in a serious light; she became subject to difficulty of deglutition. The report says, that she was quite unable to swallow any solid food, and even the passage of liquids produced considerable pain, accompanied by a choking sensation, and that the food was frequently forcibly ejected from the mouth in the effort at deglutition, and that much of it passed through the posterior nostrils.

Now, you may naturally ask, what has the larynx to do with deglutition; it is true that, in swallowing, provision is made to protect the glottis, but how can disease of the larynx create dysphagia? A very little consideration of the close connexion existing between the pharynx and the larynx, and also of the intimate relations of the nerves which supply both, will furnish the solution of this problem.

You know that the rima glottidis lies immediately behind and beneath the root of the tongue, and that the epiglottis stands up between both, and seems to protect and overhang the glottis. In deglutition, the root of the tongue and the rima glottidis are forcibly compressed together, and the epiglottis, lying between them, also suffers compression, and is made thereby to cover the whole chink of the glottis. This is the mechanism by which, in deglutition, food is prevented passing into the larynx; this close apposition of the root of the tongue to the rima glottidis serves to close the latter aperture completely, provided the epiglottis retain its normal flexible and elastic state. But if the epiglottis be swollen or thickened, and rigid, or even simply highly sensitive and irritable, as from ulcers on its surface, then, by its intervention, that perfect apposition of the root of the tongue to the glottis is prevented, on which perfect closure of the glottis, and, consequently, perfect deglutition, depend.

The epiglottis may have been removed, as in Majendie's experiments and observations; and, provided no material injury have been done to the neighboring textures, the apposition to which I allude may be effected, and the glottis protected. But it rarely happens, after chronic destructive disease of the epiglottis, that the neighboring textures have so far escaped as to allow full play to the lingual and pharyngeal muscles, so that they may perform freely, and without impediment, the actions necessary for deglutition.

Disease of the larynx, then, gives rise to difficulty of deglutition, when the epiglottis, or the arytæno-epiglottidian folds of mucous membrane, but especially the former, are involved in the disease. And the degree of dysphagia is greatest when the epiglottis is swollen or so irritable that the actions necessary for deglutition are impeded through a mechanical obstacle, or through extreme sensibility of the surface of the mucous membrane.

The nature of the dysphagia, in cases of this kind, deserves your attention. It is not only often extremely painful, and the actual effort of swallowing difficult, but the whole act of deglutition is so deranged, that the usual safeguards to the larynx below, and to the nares above, are greatly interfered with. Hence, in many instances, and especially when the epiglottis is rigid and swollen, the attempt to swallow is followed by great irritation of the glottis and by a powerful expiratory effort, by which the food or fluid is forcibly ejected upwards, partly through the mouth, and partly, and most painfully, through the posterior nares. This kind of inversion of the act of deglutition, when it frequently occurs, and is associated with other signs of laryngeal disease, is always an indication of a diseased state of the epiglottis. This feature, then, of difficult deglutition necessarily directed our attention very much to the state of the larynx in our patient.

But to proceed with the history of the case. Since the commencement of her attack, she had lost flesh considerably, and had been troubled with perspirations at night. She has frequently suffered from pain between her shoulders, and her breath has been gradually becoming more and more short. She never spat blood. As winter came on the pain returned; she lost her voice, so that she was only able to speak in a whisper, and her breathing became stridulous,—a symptom distinctly pointing to the larynx either as primarily or secondarily diseased. This symptom never disappeared; and while she was in the hospital, the noise of her breathing was so loud and peculiar, that, upon coming into the ward, your attention could not fail to be arrested by it. At the same time she suffered from a troublesome hacking cough, accompanied with the expectoration of a greenish muco-purulent matter; her deglutition became worse, and she was unable to swallow even very small quantities of liquids or solids without considerable difficulty and pain.

The first question which proposed itself for our consideration, was whether the laryngeal symptoms arose from the occurrence of certain morbid changes in the larynx itself,—in fact, were dependent upon disease of the larynx,—or whether they were caused by the pressure of some intrathoracic tumor on the left recurrent nerve. That pressure on the recurrent is quite sufficient to give rise to such symptoms, has been abundantly proved by cases of thoracic aneurism. The aneurisms which usually produce such pressure are small, globular dilatations of the vessel, occurring about the bifurcation of the trachea.

Some years ago, I recollect meeting with a case of this kind which exhibited all the more prominent symptoms of chronic laryngitis. The patient was brought into the ward just as I was leaving it after my visit, and I had no opportunity of making a sufficiently minute examination of her at that time. There were great emaciation, stridulous breathing, dyspnœa, with chronic cough, hoarseness, and pain referred to the larynx. Unfortunately, the patient died very soon after her admission, and probably in consequence of exhaustion brought on by moving her. At the *post-mortem* examination, we found an aneurism situated just at the bifurcation of the trachea, and pressing upon the left recurrent nerve so forcibly as to cause complete obliteration of the nerve tubercles; hence there was complete paralysis of the muscles of the larynx supplied by the nerve of this side, and they were found small, ill-nourished, and shrivelled.

Some months ago we had a remarkable case in Rose Ward, as to the precise nature of which we had some doubt. The man suffered from symptoms clearly referrible to the trachea and larynx. He was troubled with violent irritative cough, and the expectoration was tinged with blood; but the voice was slightly affected, and the breathing was not stridulous. The diagnosis lay between ulcerative disease of the trachea and the existence of a small aneurism pressing on the recurrent nerve. The patient died suddenly by hæmorrhage; and a little above the bifurcation of the trachea we found a small perforating ulcer, which had incidently been caused by the pressure of an aneurism of the arch of the aorta against the trachea.

How, then, are we to make the diagnosis between actual laryngeal disease and that deranged state of the larynx which stimulates inherent disease of the organ, but which really depends upon the existence of an irritating or paralysing cause at a distance from the larynx?

To determine affirmatively the existence of inherent disease of the larynx, you must not trust solely to the symptoms. Those symptoms you will find to be impaired voice, breathing difficult and stridulous, and the dyspnœa, although constant within certain limits, yet becoming much exacerbated from time to time, pain referred to the larynx, and more or less difficulty of swallowing. Now, all of these symptoms may be caused by the pressure of an aneurism or other intrathoracic tumor on the recurrent nerve.

You must add, therefore, to the examination of symptoms, inspection with the finger, which alone will often enable you to decide.—With the forefinger of the right hand you will generally be able to reach the epiglottis with great ease, and you may often feel its laryngeal surface; the finger may be passed along the arytæno-epiglottidian folds, and any thickened or roughened state of the mucous membrane covering these parts can be readily felt. When the epiglottis is much thickened, you will find it more or less rigid, with edges rounded, or it may be so swollen as to appear like a small globular tumor between the tongue and the larynx. If the mucous membrane covering the epiglottis be diseased, the surface will feel uneven or rough, or it may be hollowed out into small depressions, with irregular and perhaps callous edges. Generally, when the mucous membrane of the larynx is affected with chronic inflammation, that of the fauces is often found to sympathize with it; hence, upon looking into the mouth, you will often notice an injected state of the mucous membrane covering the back of the mouth and throat. When ulcers exist in the larynx there will usually be found a certain amount of purulent expectoration, which may in part, however, come from the lungs, if, as usually happens in cases of laryngeal phthisis, these organs are also affected with tubercular deposit. On the other hand, if the lungs be found perfectly healthy, it may be inferred that all the secretion is derived from ulcers in the larynx, which is the case in syphilitic ulceration uncomplicated with other disease. In tubercular disease, expectoration is only met with in cases where the tubercles are being softened and broken down. In the crude state, before the tubercular deposit has undergone disintegration, there is no expectoration whatever from the lungs.

If the laryngeal symptoms are caused by an intra-thoracic tumor, there can be no difficulty in the diagnosis when there is a bulging or prominence to be found in any part of the chest; but if the tumor be small, and situated near the bifurcation of the trachea, considerable difficulty will often be experienced before any conclusion can be arrived at, and in such cases the diagnosis will rest in a great degree upon negative evidence. The absence of pain referred to the larynx, and the absence of purulent secretion, will to a certain extent direct the attention to the interior of the thorax for an explanation. The degree and kind of dysphagia will sometimes help you. Generally speaking, the dysphagia is not nearly so great nor so prominent a symptom where there is intra-thoracic tumor, as in cases of laryngeal disease; and it differs also in kind. In the latter, the dysphagia is evidently obstructive, so to speak, and the food is apt to go the wrong way; it sputters back into the mouth and into the posterior nares; but in tumor cases there is a feebleness and difficulty in using the pharyngeal muscles while the passage is quite free and unobstructed.

The respiratory movements in aneurismal cases are more hurried and otherwise impaired than when the larynx only is affected, although air passes freely into the lungs, or the greater part of them. In laryngeal cases the respiratory affection depends upon the amount of obstruction which exists to the passage of air into the lungs, from the diminution of the size of the glottis; and, in these cases, the dyspnœa arises from the want of air. In these laryngeal cases, auscultation indicates feebleness of breathing and faintness of respiratory murmur, which are uniform if there be no localised tubercular deposit. In intra-thoracic tumor you may have general-rhonchus, accompanying a paroxysm of dyspnœa; or, if the tumor press on one bronchus more than another, the ronchus will be greatest on that side, or the sounds of breathing most feeble; it will be plain that less air gets into that lung than into its fellow. In the present case we had no difficulty in coming to a conclusion, the tubercular diathesis being well marked both in the patient's history, and also by the presence of physical signs; moreover, the patient's age was against the presence of aneurism, and this is a point which will often prove of valuable assistance to you in pronouncing an opinion, for aneurism very seldom occurs before the age of thirty.

In our patient, it was a question at first whether the disease of the larynx was syphilitic or tubercular. There was no history of syphilis to be obtained from the girl herself, but this, as you may easily conceive, could not be considered as conclusive against the syphilitic origin of the malady. There were, however, no other marks or symptoms of syphilis. However, there could be no doubt about the existence of tubercle. Pthisis was traced in her family history, and the upper part of the left side of chest yielded a dull sound to percussion, both in front and behind. The breathing in this situation, although very feeble, was distinctly tubular, and there was, so far as the sign could be depended on in a case where voice was at a minimum, increased resonance of voice. On the right side, in the situation of the apex of the lung, there was ronchus and some crepitation.

From all these symptoms and signs we set the case down as one of tubercular disease of the lungs, in which there was a chronic thickening of the mucous membrane of the larynx and epiglottis, and probably ulceration in or near the ventricles of the larynx, impending the movements of the chordæ vocales. Although in laryngeal cases the precise seat of the disease may generally be most accurately assigned, we cannot always predicate the particular nature of the affection, which may sometimes be merely thickening, and sometimes ulceration of the mucous membrane. I know of no definite sign which will enable us to diagnose with certainty the presence of ulceration, but it exists in a large number of cases of laryngeal disease connected with pulmonary pthisis, and, if there be blood and pus in the sputa, it will probably be always found. In tubercular ulceration, the ulcers appear to be formed by the irritation and inflammation consequent upon the deposit of tubercular matter in the follicles of the mucous membrane. At the same time, Louis holds that laryngeal and tracheal ulcerations may be caused simply by the irritation produced by the contact of the tubercular matter expectorated from the lungs, and I have more than once observed a fact which certainly seems to bear out this explanation. I have found crude tubercles in one lung, and softened tubercles in the opposite lung; the bronchus connected with the lung in which the tubercles were softened, exhibited an ulcerated state of the mucous membrane, while the bronchus of the opposite side was entirely free from them, which, no doubt, might be attributed to the passage of sputa along the one, and not along the other.

In our patient we inferred the existence of crude tubercles in the left lung, but we thought that in the apex of the right lung softening of tubercles had taken place, and that possibly a small cavity might have been formed. I thought that in this case the larynx was very likely affected with aphthous ulcerations, in their nature very similar to those aphthous ulcers which are so common on the tongue and fauces. The mucous membrane of the epiglottis felt as if it were considerably thickened, and no doubt the same condition prevailed in that covering the lips of the glottis, so that the chink became in this way much narrowed, and a considerable impediment was offered to the free entrance of free air into the lungs. On the epiglottis I thought I could detect a number of small ulcerations, more particularly on its laryngeal surface. Such ulcerations would readily increase the difficulty of deglutition and the pain which the girl suffered when anything passed over the epiglottis. The mucous membrane was so irritable, that when the patient attempted to swallow liquids, a great quantity was often ejected through the posterior nares.

The symptoms did not vary much in the further course of the case. Treatment, as you would expect, was of very little use, and all that we attempted to do was to uphold the strength with nourishing food, and to relieve the distressing pain and irritability of the throat, which prevented her from sleeping, by giving small doses of opium at night.

Occasionally, to relieve the extreme irritability of the larynx, a sponge, tied on a probang, and soaked in a strong solution of nitrate of silver, was passed down to the larynx, so as to apply the solution well to the epiglottis, and to allow some of it to trickle down into the glottis. This application was always followed by considerable relief, as the patient always expressed herself as much better after each application, and her pain was relieved, although only temporarily.

The difficulty of swallowing and the dyspnœa increased in severity, and the vomiting continued unabated, so that she was unable to take much nourishment. The exhaustion increased, and, on the 25th, she was attacked with convulsions, from which she never rallied.

In the upper lobe of the right lung, a cavity about the size of a filbert was found, and was filled with pus. The remainder of the upper lobe, of the same lung, was infiltrated with tubercular matter. The upper lobe of the left lung contained crude tubercles, so that tubercular disease was not much advanced. We found numerous aphthous ulcerations on the mucous membrane of the ventricles and cordæ vocales, and also upon the laryngeal surface of the epiglottis, and these ulcers you may now see in the preparation. The mucous membrane covering the epiglottis and upper part of the larynx was much thickened, and the glottis very much contracted in size.

In reference to the frequency with which ulceration is met with in different parts of the air passages, Louis states, that, out of seventy-one cases, there were found thirty-one in which ulcers were found in the trachea, twenty-two in which the larynx was similarly affected, and in eighteen ulcers were found upon the epiglottis.

I shall now notice another case, which is more deserving of your attention than the last, inasmuch as it is an example of a very common affection of the fauces and larynx, and one which is curable, or at least very manageable. The patient is a man named Osborne, in Sutherland Ward. His symptoms are a harsh, irritative cough, with slight mucous expectoration, in quantity not at all proportionate to the violence of the cough, and also a considerable degree of hoarseness of voice. Upon looking into his mouth you find the mucous membrane of the faucial region exhibiting a dusky red blush, and you will observe a number of red points, as of raised papillæ, which are the mucous glands of the velum and back of the pharynx, in an enlarged and swollen state. The appearance of the mucous membrane generally, was one of great laxity, and the uvula was more or less elongated. In some cases the uvula is so much increased in length that it reaches to the glottis, and excites irritative cough. The inflammation upon which this state of mucous membrane depends, never leads to the formation of pus or lymph. It may, however, run into a slightly œdematous state, but this is rare; and it is not always limited to the pharynx only, but often extends to the larynx and trachea, and sometimes into one or more bronchial tubes. This kind of inflammation is very common in men of gouty diathesis, and in women of a relaxed habit who do not take proper care of their health. Such persons you will often find complaining of being very subject to attacks of hoarseness, and liable to catch cold upon the slightest exposure, and even without any apparent cause. The hoarseness will remain after the other symptomps of the cold have gone for a considerable period, in spite of various forms of treatment adopted for the cure, and it is accompanied with a troublesome cough which harasses the patient very much. Persons laboring under such symptoms as these are often treated for bronchitis, and take large quantities of expectorant and other medicines for the relief of the cough. The seat of the irritation upon which the cough depends is thought to be in the bronchial tubes, and its real position (the fauces) is overlooked. On carefully examining a patient laboring under this affection, you will find the lungs quite sound and the bronchial tubes free from irritation. Such being the case, you next proceed to examine the fauces, and you find the swollen, red, relaxed condition of membrane which I have described.

The character and constituents of the cough will help you to distinguish this affection. It is a highly irritating cough; the patient coughs with all his might to dislodge something which irritates the fauces or the larynx and upper part of the trachea. The product of the cough is very trifling, a little saliva and mucous, or throat and nasal mucous, which in London is often mixed with sooty matter. The expectoration is in general infinitely small as compared with the vehemence of the cough. Exposure to cold air always excites and greatly aggravates the cough. The patient often complains that his cough is particularly troublesome on his first going to bed; this may be either from change of temperature from warm to cold, or it may be caused by the assumption of the horizontal position, when the uvula dropping upon the glottis may excite cough.

Cases of this kind are most rife during the cold winter months, and in the early spring, when the cold north or east winds prevail so much.

With regard to our patient Osborne, he was a hard-working, industrious man, with somewhat of the lithic acid diathesis. Three years ago he was admitted into hospital with several small, hard tumors in the tongue, each about the size of a marble, which excited our fears as regards their malignant nature. We were not able to determine any very satisfactory history of syphilis, but they disappeared very quickly under iodide of potassium, and he got perfectly well. In the beginning of this winter, however, he was attacked by cough, which he attributed to exposure to cold. He had been working hard all day in a close room, and in the evening was exposed to the cold air on his return home. This soon brought on irritative cough, which was very obstinate, and did not yield to the usual remedies. On carefully examining the chest, we found no indications of bronchial irritation, but the fauces presented the injected, swollen, relaxed condition of mucous membrane, with enlarged mucous glands, which I have already described.

I treated him with the local application of the solution of nitrate of silver (ʒss. to the ℥j.) by means of a probang, which was thrust behind the epiglottis, down to the glottis, on the plan of Dr. Horace Green, of New York. The patient can always tell whether the sponge enters the larynx or not, from the great irritation it excites when it passes into the glottis; and in the withdrawal of it the operator feels a certain resistance, caused by the sponge being grasped by the muscles of the larynx, which resistance is not felt when it simply passes into the œsophagus. To pass the sponge into the larynx requires a good deal of steadiness and expertness on the part of the operator. While I fully admit the feasibility of the operation, I nevertheless suspect that the sponge may often pass simply into the œsophagus when it is thought to enter the larynx.

The application was continued every morning for three weeks, either to the glottis or to the neighboring mucous membrane; and partly, no doubt, from this cause, and partly from his avoiding exposure to the cold air, he left the hospital very much relieved, at the expiration of that period.

This case affords a good example of that particular form of affection of the mucous membrane of the throat and larynx which is not benefitted by the administration of any drug whatever, but which almost always is relieved by the local application of nitrate of silver, sulphate of copper, or even of simply astringent substances.

This plan of treating affections of this kind has long been familiar to practical men in this country, and was long ago practised very exten sively by the late Mr. Vance, of this city. Dr. Green, of New York, had the boldness to pass the sponge into the larynx, and to show that such an operation was a less formidable one than was previously supposed. It is, however, an operation not wholly free from danger, and which is not attended with proportionately good results. I do not hesitate to state this from considerable experience of it. In the vast majority of cases, quite as good effects may be obtained from applying the solution to the neighboring mucous membrane. Pass the probang down to the glottis, and swab well about its neighborhood, and you will do as much good as if you passed the sponge into the rima glottidis; and sometimes you will do more good and cause less irritation.

For some years past I have been in the habit of applying the solid nitrate of silver to the mucous membrane of the fauces, the velum, uvula, and the pillars of the palate; and it may be brought very near to the laryngeal membrane by sliding the caustic along the posterior pillars of the palate, some way down. By this treatment you may obtain results quite as satisfactory as by pushing the probang into the glottis, and in many instances more so; and the plan is, I think, on the whole, safer and more manageable.

I have been supplied by Mr. Matthews, the surgical instrument-maker of Portugal street, with a modification of the ordinary porte-caustique, which is very useful for applying nitrate of silver to the throat.

The caustic is placed in a case made of platina; this moves on a ball-and-socket joint, and may, by that means, be fixed at any angle. Its handle is constructed in telescope fashion, and may be drawn out to any length that can be required; so that, by its aid, you may apply the caustic very low down.

But in the application of nitrate of silver a great deal of caution is necessary. You must take great care not to apply it too freely, else you may cause too much inflammation and ulceration. In some cases, indeed, it is impossible to avoid these consequences; but, with due care, you need never find them so much as to be troublesome, and very often they are salutary. I always make the patient use the precaution of gargling his throat very frequently with the coldest water—iced water if it can be had—for some hours after the application of the caustic; and by these means inflammation is limited, and the parts strengthened.

If time permitted, I could tell you of numerous instances of coughs of the most troublesome kind, and of long duration, which had resisted all the ordinary cough medicines, and yielded to three or four applications of nitrate of silver.—*Lon. Med. Times and Gaz.*

